# Using an appetitive operant conditioning paradigm to screen rats for tinnitus induced by intense sound exposure: Experimental considerations and interpretation

**DOI:** 10.3389/fnins.2023.1001619

**Published:** 2023-02-10

**Authors:** Sarah H. Hayes, Krystal Beh, Marei Typlt, Ashley L. Schormans, Daniel Stolzberg, Brian L. Allman

**Affiliations:** ^1^Department of Anatomy and Cell Biology, Schulich School of Medicine and Dentistry, The University of Western Ontario, London, ON, Canada; ^2^National Centre for Audiology, Elborn College, The University of Western Ontario, London, ON, Canada; ^3^Audifon GmbH & Co. KG, Kölleda, Germany; ^4^Department of Physiology and Pharmacology, Schulich School of Medicine and Dentistry, The University of Western Ontario, London, ON, Canada

**Keywords:** tinnitus, animal models, two-alternative forced-choice behavior, intense sound exposure, hearing loss

## Abstract

In an effort to help elucidate the neural mechanisms underlying tinnitus in humans, researchers have often relied on animal models; a preclinical approach which ultimately required that behavioral paradigms be designed to reliably screen animals for tinnitus. Previously, we developed a two-alternative forced-choice (2AFC) paradigm for rats that allowed for the simultaneous recording of neural activity at the very moments when they were reporting the presence/absence of tinnitus. Because we first validated our paradigm in rats experiencing transient tinnitus following a high-dose of sodium salicylate, the present study now sought to evaluate its utility to screen for tinnitus caused by intense sound exposure; a common tinnitus-inducer in humans. More specifically, through a series of experimental protocols, we aimed to (1) conduct sham experiments to ensure that the paradigm was able to correctly classify control rats as not having tinnitus, (2) confirm the time course over which the behavioral testing could reliably be performed post-exposure to assess chronic tinnitus, and (3) determine if the paradigm was sensitive to the variable outcomes often observed after intense sound exposure (e.g., hearing loss with our without tinnitus). Ultimately, in accordance with our predictions, the 2AFC paradigm was indeed resistant to false-positive screening of rats for intense sound-induced tinnitus, and it was able to reveal variable tinnitus and hearing loss profiles in individual rats following intense sound exposure. Taken together, the present study documents the utility of our appetitive operant conditioning paradigm to assess acute and chronic sound-induced tinnitus in rats. Finally, based on our findings, we discuss important experimental considerations that will help ensure that our paradigm is able to provide a suitable platform for future investigations into the neural basis of tinnitus.

## 1. Introduction

Tinnitus is the subjective perception of a phantom sound that is often described as a ringing or buzzing sensation in the ears. In the majority of cases, tinnitus is experienced temporarily, with the phantom auditory perception fading within a few minutes or hours (Henry et al., 2005). However, for as many as 10–15% of the general population, tinnitus is experienced chronically, with 1% of the population having severely debilitating forms of tinnitus that negatively impact their daily lives (Heller, 2003). Despite decades of research, there is still no widely accepted treatment available that readily suppresses tinnitus, in part because the underlying neural mechanisms responsible for this phantom perception remain elusive. Further insight into the pathophysiology of tinnitus is expected to rely heavily on animal studies involving neural recordings; an approach which first requires that researchers be able to reliably screen animals for the presence/absence of tinnitus. For a behavioral paradigm to be most effective, it should be able to (1) screen for both acute and chronic tinnitus, (2) closely reflect the human condition, (3) be able to account for the presence of hearing loss associated with tinnitus induction methods (e.g., hearing loss associated with noise exposure), and (4) allow for individual comparisons to address variability amongst tinnitus sufferers (Hayes et al., 2014).

Many of the existing behavioral paradigms used to screen animals for tinnitus are based on one of three general methods: shock avoidance (Jastreboff et al., 1988; Bauer et al., 1999; Bauer and Brozoski, 2001; Heffner and Harrington, 2002; Guitton et al., 2003; Rüttiger et al., 2003; Lobarinas et al., 2004; Yang et al., 2011; Pace et al., 2016; Jones and May, 2017; Zuo et al., 2017), appetitive two-choice operant conditioning (Sederholm and Swedberg, 2013; Stolzberg et al., 2013), or gap prepulse inhibition of the acoustic startle response (GPIAS; Turner et al., 2006). As noted in recent review articles on the topic (Hayes et al., 2014; Galazyuk and Brozoski, 2020), although each of these paradigms has its advantages, there are also notable challenges that can detract from their effectiveness as a screening tool for tinnitus. For example, some traditional shock avoidance paradigms present the issue of behavioral extinction, which precludes the ability to study persistent forms of tinnitus. Additionally, appetitive two-choice operant conditioning models can be limited by the extensive period required to train the animals prior to actually performing tinnitus screenings. Consequently, the GPIAS paradigm—which does not require overt training—quickly became the most popular behavioral method used to screen animals for tinnitus due to its high-throughput nature. However, recent studies have highlighted the need to be cautious when interpreting GPIAS results due to the potential confound of screening hearing-impaired animals for gap detection deficits using a metric reliant on their acoustic startle reflex (Longenecker and Galazyuk, 2011, 2012, 2016; Lobarinas et al., 2013; Longenecker et al., 2018), as well as the fact that the GPIAS paradigm and similar gap detection tasks have yet to convincingly identify tinnitus in human subjects (Campolo et al., 2013; Fournier and Hébert, 2013; Boyen et al., 2015).

Preclinical investigations into the neural basis of tinnitus can benefit from combining a behavioral screening with simultaneous neurophysiological recordings at the very moments when the animals are attending to their tinnitus; an approach consistent with human testing. To achieve our goal of recording neural activity as rats actively reported behavioral evidence of tinnitus, we previously designed a two-alternative forced-choice (2AFC) appetitive conditioning paradigm that required rats to categorize whether they were hearing either steady narrowband noises (NBNs), an amplitude-modulated (AM) broadband noise, or quiet (Stolzberg et al., 2013). As we were motivated to design our 2AFC task to be compatible with recording tinnitus-related cortical oscillations—the synchronized neural activity that has been suggested to underlie phantom perception (Weisz et al., 2005, 2007a,b; for review see Adjamian, 2014)—the rats were trained to poke their nose in a central port and hold relatively still for several seconds while attending to the stimulus condition (NBNs, AM or quiet) that was being presented on a given trial. During the quiet trials, this holding period would provide a sufficient epoch to accurately record the low-frequency oscillations implicated in tinnitus pathophysiology (Adjamian, 2014). Following the holding period, a cue light signaled to the trained rats to nose-poke in one feeder trough for NBNs and the other feeder trough for both AM noise and quiet trials. Thus, trained rats would go on to screen positive for tinnitus if they incorrectly identified quiet trials as though they were hearing a steady NBN; findings consistent with humans who report tinnitus as the perception of persistent sound during quiet conditions. The paradigm included the AM noise trials to ensure that rats with tinnitus continued to have reason to select both feeder troughs throughout the session, regardless of whether they made correct or incorrect choices during the quiet trials. To validate the effectiveness of the paradigm, rats were exposed to a high dose of sodium salicylate, which is known to reliably induce transient tinnitus in rodents and humans (Mongan et al., 1973; Jastreboff et al., 1988; Cazals, 2000; Guitton et al., 2005; Lobarinas et al., 2006). As predicted, rats that were able to correctly identify the quiet trials during a control session (saline injection), now went on to screen positive for tinnitus in the hours following sodium salicylate injection because they reported hearing steady NBN during a significant number of quiet trials (Stolzberg et al., 2013). Furthermore, the electrophysiological recordings made during the behavioral testing revealed that the aberrant cortical oscillations observed in rats experiencing salicylate-induced tinnitus largely paralleled the findings reported in tinnitus patients (Weisz et al., 2005).

Although our above-mentioned 2AFC behavioral paradigm showed great promise as a way to reveal the neural changes associated with salicylate-induced tinnitus, its capacity to reliably screen rats for tinnitus caused by intense sound exposure would need further evaluation. Thus, in the present study, we assessed the utility of our 2AFC behavioral paradigm to screen for intense sound-induced tinnitus by (1) conducting sham experiments to ensure that the paradigm was able to correctly classify control rats as *not* having tinnitus, (2) confirming the time course over which the behavioral testing could reliably be performed post-exposure to assess chronic tinnitus, and (3) considering if the paradigm was sensitive to the variable outcomes often observed after intense sound exposure (e.g., subjects with considerable hearing loss but without tinnitus, versus those with tinnitus and only limited hearing loss). In the first experimental series, we exposed rats to intense sound or sham conditions for 15 min, and immediately screened them for acute tinnitus using our 2AFC behavioral paradigm, with the expectation that no rats should falsely-screen positive for tinnitus post-sham exposure, yet all rats would show behavioral evidence of acute tinnitus immediately after the 15-min sound exposure. Next, in preparation to study persistent tinnitus, pilot experiments were conducted on a separate cohort of control rats to determine how many weeks could elapse as well as how many test sessions could be performed repeatedly before the behavioral paradigm failed to accurately report the *absence* of tinnitus. Based on these findings, we then used our 2AFC behavioral paradigm to screen for tinnitus that persisted one week after intense sound exposure, with the expectation that there would be considerable variability across animals, such that not all rats would show behavioral evidence of chronic tinnitus nor have the same degree of permanent hearing impairment. Overall, the present study documents the utility of our appetitive operant conditioning paradigm to assess acute and chronic sound-induced tinnitus in rats; an important step in moving toward using this paradigm to simultaneously record neural activity during the behavioral screening for tinnitus induced by intense sound exposure.

## 2. Materials and methods

A total of 23 adult male Sprague-Dawley rats (Charles River Laboratories, Inc., Wilmington, MA), separated into three experimental series, were used in the present study. All rats (60 days old at the onset of training), were housed in a 12-h light-dark cycle with water *ad libitum*. All experimental procedures were approved by the University of Western Ontario Animal Care and Use Committee and were in accordance with guidelines established by the Canadian Council of Animal Care.

### 2.1. Behavioral apparatus

The behavioral apparatus consisted of a standard modular test chamber (ENV-008CT; Med Associates Inc., St. Albans, VT, USA) housed in a sound-attenuating box (29” W by 23.5” H by 23.5” D; Med Associates Inc.). The front wall of the behavioral chamber included a center port with two stainless steel feeder troughs positioned on either side; each fitted with an infrared (IR) beam used to detect nose-pokes. Each feeder trough was attached to a food pellet dispenser located behind the behavioral chamber. A house light was located on the back wall to illuminate the chamber, and a white light-emitting diode (LED) was located directly above the center nose-poke, which served as a GO cue during behavioral training. Real-time processing hardware (RZ6 and BH-32, Tucker Davis Technologies, Alachua, FL) were interfaced with the test chamber. Custom behavioral protocols running in Matlab (EPsych Toolbox, dstolz.github.io/epsych/) monitored the nose poke responses, and controlled the presentation of the auditory stimuli, as well as the positive reinforcement (i.e., food pellet delivery) and punishment (i.e., the inability to begin the next trial during a 15-s timeout period, indicated by turning off the house light).

Acoustic stimuli were programmed to play from a speaker (FT28D; Fostex, Tokyo, Japan) mounted on the ceiling of the behavioral chamber. There were three types of acoustic stimuli used in the paradigm: quiet (speaker off), amplitude-modulated noise (AM; broadband noise, 100% modulation, 5 Hz), or one of five narrowband noises (NBN; 1/8th octave band, center frequencies at 8, 12, 16, 20, or 24 kHz). One of the acoustic stimuli conditions was always present in the behavioral chamber regardless of trial initiation by the rat. AM and NBN stimuli were calibrated using TDT hardware (RPvdsEx, RZ6 module; TDT) and custom MATLAB software (Mathworks) to ∼75 dB sound pressure level (SPL) using a 1/4” microphone (2530, Larson-Davis, Depew, NY, USA) and pre-amplifier (2221, Larson-Davis).

### 2.2. Behavioral training

**FIGURE 1:**
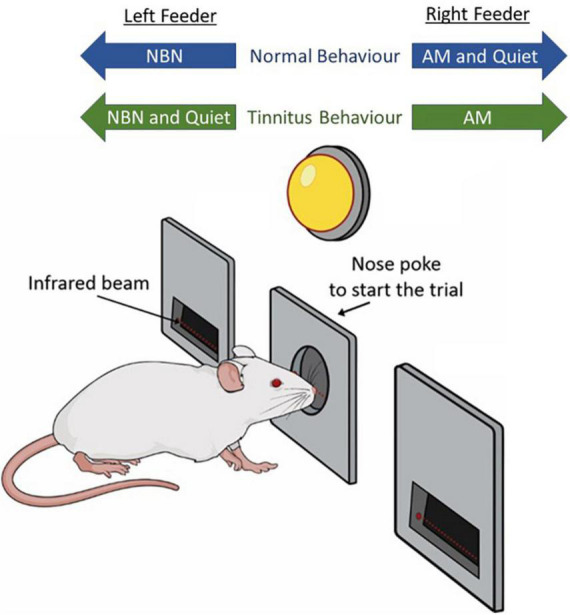
Schematic representation of behavioral paradigm. Rats hold their nose in a center port until an LED flashes, which serves as a GO cue. They are then trained to access the left feeder trough during narrowband noise (NBN) trials, and the right feeder trough for amplitude-modulated (AM) broadband noise and Quiet trials. Following tinnitus induction via intense sound exposure, rats experiencing tinnitus are expected to respond to the NBN (left) feeder trough during Quiet trials, indicating they perceived a steady phantom sound during quiet conditions.

Prior to commencing behavioral training, rats were food restricted to ∼85% of free-feeding weight to encourage exploration in the behavioral boxes. Rats were trained 30 min per day, and 6 days per week. Behavioral training progressed through a series of steps described in Supplementary Table 1. Initial training sessions (Phase 1) required rats to nose-poke a center port (detected by interruption of the center IR beam) to trigger a GO cue (flash of LED) (Figure 1). Upon removing its nose from the center port, the rat was immediately reinforced with a food pellet (Bio-Serv, Frenchtown, NJ, USA) which was dropped into the appropriate feeder trough associated with the acoustic stimulus playing from the overhead speaker; i.e., left feeder trough for 16 kHz NBN, and right feeder trough for quiet. If the rat then nose-poked the correct feeder trough within 5 s of the initial pellet delivery (detected by the interruption of the trough IR beam), the rat was given a second food pellet to further reinforce the stimulus association. During a 30-min training session, trial type (16 kHz NBN or quiet) was distributed evenly and presented in a randomized order. As rats became more proficient at the task, the cue delay (time required to trigger the GO cue) was progressively increased from 500 to 2,500 ms.

Upon learning to frequently nose poke the center port (typically after 2 to 3 days), rats were then trained on a new protocol (Phase 2A) where the initial pellet reinforcement was removed and pellet delivery was provided only if the rat poked its nose in the correct feeder trough in response to the given acoustic stimulus. Rats received 100% reward rates, and throughout all phases of training, incorrect feeder trough responses were punished with a 15-s timeout during which time the next trial could not be initiated. Rats remained on Phase 2A until they could correctly associate feeder troughs with the given acoustic stimuli with >92% accuracy for at least three consecutive days (typically after two weeks).

Once rats could correctly distinguish quiet trials from 16 kHz NBN trials, a new protocol (Phase 2B) was introduced where rats were trained to nose poke the right trough for quiet trials, and the left trough for all NBNs (8, 12, 16, 20, or 24 kHz). Rats continued to receive 100% reward rates for correct responses. Trial type (NBN or quiet) was distributed evenly and presented in a randomized order. Upon learning the correct feeder trough associations for at least five consecutive days at >92% accuracy (typically after two weeks), rats were trained on a new protocol (Phase 2C) where the left feeder trough represented all NBN trials, and the right feeder trough represented quiet and AM trials. During a 30-min training session, 50% of trials were NBN, 30% of trials were AM, and 20% of trials were quiet; trials were presented in a randomized order according to criteria provided by Gellermann (1933). Rats continued to receive 100% reward rates for correct responses, and timeouts for incorrect responses. Once rats learned the correct feeder trough associations for all three stimulus types (typically after 1 month), reward rates were progressively lowered to 70% until the rats were able to consistently achieve a > 92% hit-rate during each training session. Using this strategy, daily behavioral performance was highly consistent across all trial types (see Figure 5A from Stolzberg et al., 2013).

**FIGURE 2:**
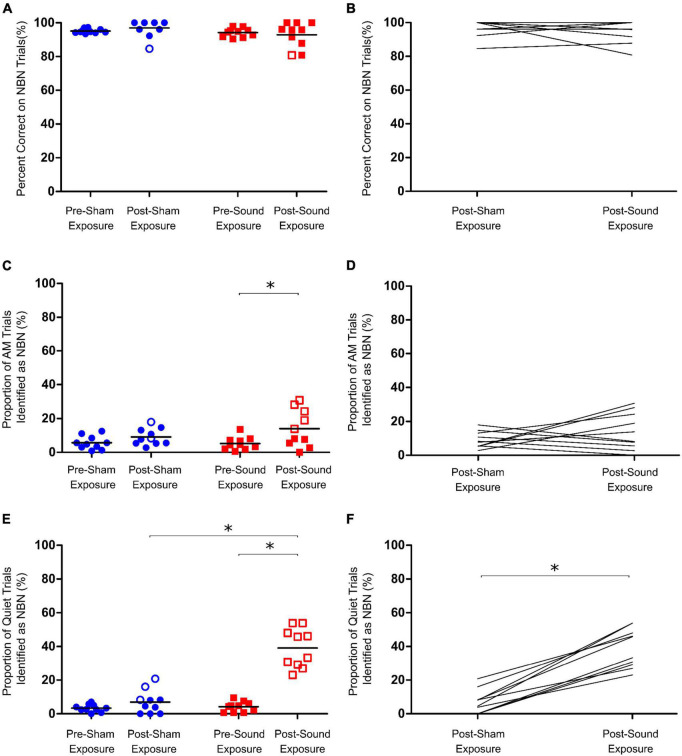
Assessment of acute tinnitus induced by intense sound exposure. **(A)** Following sham and sound exposures, rats could still accurately identify lower frequency narrowband noise (NBN) stimuli. **(B)** No change in NBN performance was observed between post-sham and post-sound exposure conditions. **(C)** AM trial performance was unaffected by sham exposure; however, at the group level, a significant increase in misidentification of AM trials as NBN was observed post- sound exposure. **(D)** No change in AM performance was observed between post-sham and post-sound exposure conditions. **(E)** Following sham exposures, rats could still correctly identify quiet stimuli. In contrast, following intense sound exposures, all rats mistakenly identified significantly more Quiet trials as NBN, indicative of tinnitus-like behavior. **(F)** On average, rats mistakenly identified significantly more Quiet trials as NBN following sound exposure than they did following sham exposure. Statistical analyses included a two-way repeated measures ANOVA (time x exposure), followed by *post hoc* paired t-tests with Bonferroni corrections, *p < 0.01, *n* = 10. Open symbols in panels **(A,C,E)** represent individual rats with z-scores exceeding the one-tailed criterion for significance (see section “2. Materials and methods” for details). In panels **(B,D,F)**, each line represents an individual rat’s performance on NBN **(B)** AM **(D)** or Quiet **(F)** trials.

**FIGURE 3:**
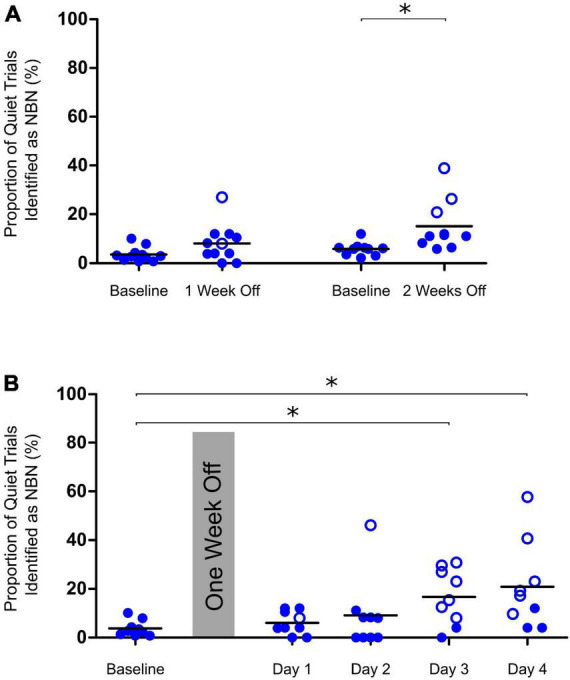
Determining appropriate time points for testing of chronic tinnitus. **(A)** Performance on Quiet trials remained consistent after control rats were given one week off between training and testing on the behavioral paradigm. After two weeks off, a significant increase in the misidentification of Quiet trials (indicating a false-positive screening of tinnitus) was observed [two-tailed paired t-test on baseline vs two weeks off, *t*(9) = 2.7, *p < 0.05, *n* = 10]. **(B)** After one week off between training and testing, control rats could be tested up to two days in a row before a significant increase in misidentification of Quiet trials was observed on days 3 and 4 of repeat testing (One-way ANOVA, *F*_4,40_ = 3.39, *p < 0.05, *n* = 9). Open symbols in panels **(A,B)** represent individual rats with z-scores exceeding the one-tailed criterion for significance (see section “2. Materials and methods” for details).

**FIGURE 4:**
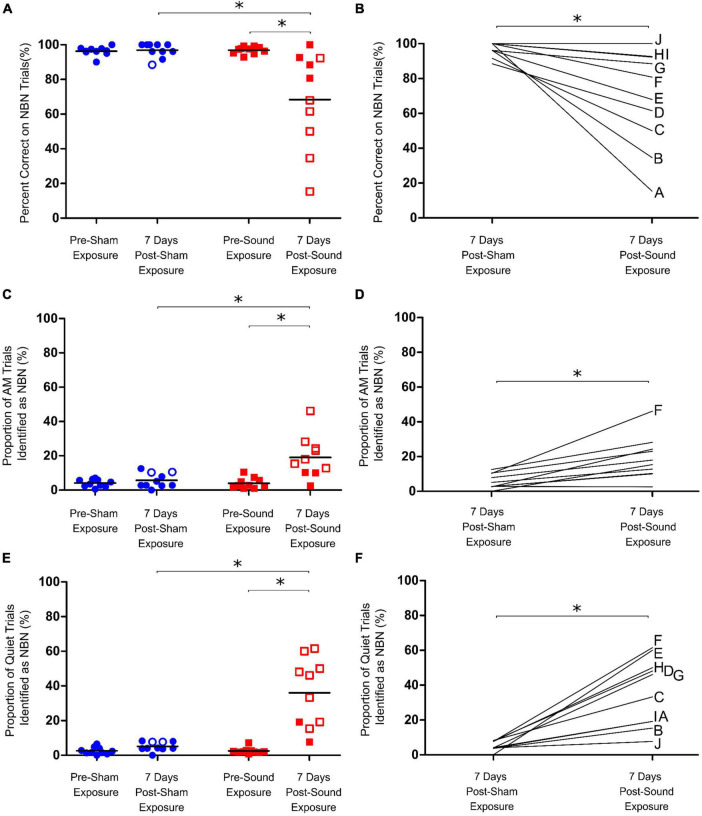
Assessment of chronic tinnitus induced by intense sound exposure. **(A)** Following sham exposure rats could still accurately identify lower frequency narrowband noise (NBN) stimuli. Following sound exposure, however, a significant drop in NBN performance was observed for the group average. **(B)** Post-sound exposure, rats misidentified significantly more NBN trials compared to post-sham, with a wide range in NBN performance observed post-sound exposure. **(C)** AM trial performance was unaffected by sham exposure; however, a significant increase in misidentification of AM trials as NBN was observed post-sound exposure for the group average. **(D)** Post-sound exposure, rats misidentified significantly more AM trials compared to post-sham. **(E)** Following sham exposures, rats could still correctly identify quiet stimuli. In contrast, following sound exposures, not all rats screened positive for tinnitus-like behavior by demonstrating an increase in the percentage of Quiet trials misidentified as NBN (i.e., closed vs. open red squares). **(F)** As a group, the rats mistakenly identified significantly more quiet trials as NBN following sound exposure than they did following sham exposure. Statistical analyses included a two-way repeated measures ANOVA (time x exposure), followed by *post hoc* paired t-tests with Bonferroni corrections, *p < 0.01, *n* = 10. Open symbols in panels **(A,C,E)** represent individual rats with z-scores exceeding the one-tailed criterion for significance (see Methods for details). In panels **(B,D,F)**, each line represents an individual rat’s performance on NBN **(B)**, AM **(D)** or Quiet **(F)** trials, with each rat identified by a separate letter on the right-edge of the graphs.

**FIGURE 5:**
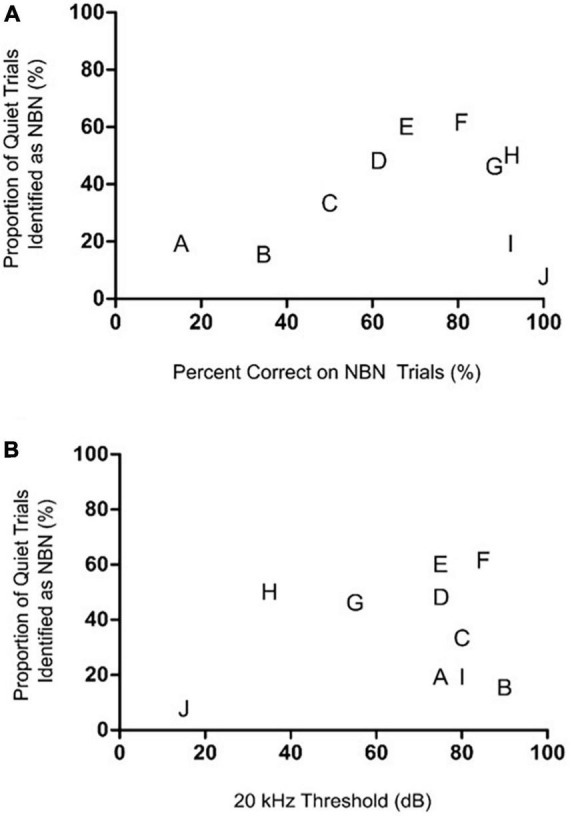
The rats’ degree of high frequency hearing loss or their ability to detect the steady NBN failed to predict their Quiet trial performance during the chronic tinnitus assessment. **(A)** Relationship between NBN and Quiet trial performance post-sound exposure during the chronic tinnitus assessment. When each rat’s post-sound exposure performance on NBN trials was plotted versus their Quiet trial performance, no correlation was observed, demonstrating that a rat’s Quiet trial performance was independent of its NBN trial performance [*r*(8) = 0.42, *p* > 0.05]. **(B)** Relationship between the post-sound exposure 20 kHz hearing threshold and Quiet trial performance during the screening for chronic sound-induced tinnitus. No correlation was observed between hearing thresholds at 20 kHz post-sound exposure and performance on Quiet trials [*r*(8) = 0.28, *p* > 0.05]. In both graphs, each rat is identified by a separate letter which corresponds to its designation in Figure 4, as this allows for visual comparisons to be made across these correlative analyses and the sham versus sound exposure results in Figure 4.

### 2.3. Behavioral testing

To screen for behavioral evidence of tinnitus, trained rats were run on a *testing protocol* in which the previously described training protocol was modified such that responses during quiet trials were no longer rewarded nor punished, in an effort to avoid biasing test day results. Rats experiencing tinnitus were expected to perceive a steady phantom sound during quiet conditions, and as such, they would more frequently respond to the left (NBN) feeder trough (previously an incorrect response) during quiet trials, rather than the right (quiet and AM) feeder trough (previously a correct response; Figure 1). During testing, reward rates were increased from 70 to 90% for NBN and AM noise trials to compensate for the lack of food pellets delivered during quiet trials. As a result, the overall reward rate was similar to that of the final training protocol.

Prior to screening rats for chronic tinnitus following intense sound exposure, we carried out pilot experiments in a cohort of animals in order to determine the appropriate time course for running the testing protocol after tinnitus induction. We determined the number of days (i.e., one or two weeks) that rats could refrain from daily training and still perform the behavioral task to criteria when subsequently run on the testing protocol, as well as how many testing days in a row they could be run on the testing protocol. These control experiments allowed us to select an appropriate time point for assessing the presence of chronic tinnitus in the absence of a confounding influence of increased durations between training and testing days. Following the completion of the pilot experiments, a time point of one week was selected as the duration between intense sound exposure and the assessment of chronic tinnitus.

### 2.4. Intense sound exposures

In the first experimental series, following three consecutive days of normal behavioral training at hit-rates of > 92% accuracy, trained rats (*n* = 10) were placed in a sound-attenuating chamber and subjected to either a 15-min sham exposure (quiet, speaker off), or a 15-min sound exposure (bilateral, 12 kHz tone, 110 dB SPL) from a super tweeter (T90A; Fostex) positioned above the home cage. Immediately after the exposure, rats were placed in the behavioral box and run on the aforementioned testing protocol for 120 to 130 trials. Between the sham and sound exposures, rats were given a minimum of five standard training days, during which time they had to consistently perform with >92% accuracy.

In a separate experimental series, trained rats (*n* = 10) were used to identify the presence of chronic tinnitus induced by intense sound. Following three consecutive days of training in which the rats demonstrated hit-rates of > 92% accuracy, they were anaesthetized with an intraperitoneal injection of ketamine (80 mg/kg) and xylazine (5 mg/kg). Once the rat’s pedal reflex was absent, it was placed on a homeothermic heating pad (maintained core temperature at ∼37^o^C; model 507220F; Harvard Apparatus) in a sound-attenuating chamber (29” W by 23.5” H by 23.5” D; Med Associates Inc.) and given a 60-min sham exposure (quiet, speaker off). Supplemental doses of ketamine/xylazine were administered intramuscularly as needed. Following the 60-min exposure, anaesthesia was reversed using an intraperitoneal injection of atipamezole hydrochloride (1 mg/kg), and the rat was returned to its home cage for recovery. Rats were not trained for the six days following the sham exposure. One week after the sham exposure, rats were run on the aforementioned testing protocol. Rats were given a minimum of five standard training days following the 60-min sham exposure test session before being prepped for the 60-min sound exposure. Once each rat had demonstrated three consecutive days of normal training at > 92% accuracy after their post-sham testing, they were again anaesthetized and placed in the sound-attenuating chamber. This time, rats were given a 60-min sound exposure (bilateral, 12 kHz tone, 120 dB SPL) from a super tweeter (T90A; Fostex) placed directly in front of their head, 5 cm from the pinna of the ears. The exposure was generated with TDT software and hardware (RPvdsEx, RZ6 module; TDT). Following the exposure, rats were administered an intraperitoneal injection of atipamezole hydrochloride (1 mg/kg) and returned to their home cage. Similar to the 60-min sham exposure, rats were not trained for the six days following the sound exposure. One week later (on Day 7), rats performed the testing protocol to screen for behavioral evidence of chronic tinnitus.

It is possible that during the initial training phases of the 2AFC task (i.e., well before the subsequent testing protocol to screen for behavioral evidence of tinnitus), the rats experienced some brain plasticity associated with first learning the rules of the behavioral protocol. However, because any learning-induced plasticity would have occurred in both the sham and tinnitus-induced rats and it would have taken place long before they underwent sham or tinnitus induction exposures, it is not expected that learning-induced plasticity would interfere with tinnitus pathophysiology while animals are run on the testing protocol of the 2AFC task.

### 2.5. Detection of hearing thresholds using auditory brainstem responses

At the conclusion of behavioral testing, hearing thresholds of rats were determined using the auditory brainstem response (ABR) to verify the extent of hearing loss in the week following the 60-min sound exposure. Rats were again anaesthetized with intraperitoneal injections of ketamine (80 mg/kg) and xylazine (5 mg/kg) and placed on a homeothermic heating pad (maintained core temperature at ∼37^o^C) in a sound-attenuating chamber (29” W by 23.5” H by 23.5” D; Med Associates Inc.). Once their pedal reflex was absent, subdermal electrodes (27G; Rochester Electro-Medical, Lutz, FL, USA) were positioned at the vertex (active electrode), over the right mastoid (reference electrode), and on the mid-back (ground electrode). Electrodes were connected to a low-impedance headstage (RA4LI; TDT), and auditory-evoked activity was preamplified and digitized (RA16SD Medusa preamplifier; TDT) prior to being sent to an RZ6 module (TDT) via a fiber optic cable. Signals were bandpass filtered (300–3,000 Hz) and averaged using BioSig software (TDT). Briefly, acoustic stimuli consisted of a click (0.1 ms), 4 kHz tone, and 20 kHz tone (5 ms duration, 1 ms rise/fall time) presented from a speaker positioned 10 cm from the rat’s exposed right ear (the left ear was occluded with a custom foam ear plug). Stimuli were each presented 1,000 times (21 times per second) at decreasing sound intensities from 90 to 10 dB SPL in 5 to 10 dB steps. Close to ABR threshold, stimuli were repeated in order to confirm an accurate threshold judgement using the criteria of just noticeable deflection of the averaged electrical activity within the 10 ms window (Popelar et al., 2008; Schormans et al., 2017, 2018). All acoustic stimuli were calibrated using a 1/4” microphone (2530; Larson-Davis), a pre-amplifier (2221; Larson-Davis), and custom MATLAB software (Mathworks).

### 2.6. Statistical analysis and data presentation

As in our previous publication (Stolzberg et al., 2013), tinnitus-like behavior was defined as a significant decrease in responses to the correct feeder trough during quiet trials on testing day compared to the response rate on quiet trials during the preceding 5 baseline days. Similarly, performance on AM and lower frequency (8–12 kHz) NBN trials was also monitored and compared to baseline performance. Lower frequency NBN trials were selected for evaluation under the assumption that they would be less likely to be affected by hearing loss following the high frequency sound exposures used to induce tinnitus. To evaluate behavioral performance on the individual level, we calculated z-scores for each rat based on its performance over the preceding 5 baseline days for each acoustic stimuli separately, and used a one-tailed criterion of *p* < 0.01 to indicate a significant change in z-score for each stimulus (Heffner, 2011; Stolzberg et al., 2013). Statistical analyses were also conducted on group behavioral data using either a two-way repeated measures analysis of variance (ANOVA), one-way repeated measures ANOVA, or paired t-test, depending on the comparison of interest (see “3. Results” section for the details of each specific comparison). All statistical comparisons used an alpha value of 0.05. When a two-way ANOVA was used, *post hoc* testing was performed with Bonferroni post-tests to correct for multiple comparisons. When a one-way ANOVA was used, *post hoc* testing was performed with Dunnett’s post-tests to compare back to baseline. Sigma Stat 3.5 was used for all statistical analyses and BioRender (Biorender.com) was used for methodology schematics. All results are presented as mean ± standard error of the mean (SEM).

## 3. Results

### 3.1. Acute tinnitus induced by brief yet intense sound exposure

To determine the ability of the 2AFC behavioral paradigm to screen for acute tinnitus in the minutes following intense sound exposure, rats underwent behavioral training to distinguish between quiet, AM noise, and NBN stimuli. Once trained, they were given 15-min sham and sound exposures immediately prior to behavioral testing to determine if either exposure resulted in behavioral performance consistent with the presence of tinnitus. Tinnitus-positive behavior was scored as a shift in the response to quiet stimuli from the right trough (previously trained to be a correct response) to the left trough (previously trained to be associated with NBNs; see Figure 1). Performance on AM noise and NBN trials was also monitored.

Following 15-min sham and sound exposures, rats were still able to correctly identify > 90% of lower frequency NBN trials, and demonstrated no significant change in NBN performance from baseline regardless of exposure type (Figures 2A, B). Similarly, although a significant increase in the number of AM trials identified as NBN was observed post-sound exposure, the rats still correctly identified > 85% of the AM trials (Two-way RM ANOVA, significant main effect for time, *F*_1,9_ = 12.902, *p* < 0.01) (Figures 2C, D). Taken together, these results demonstrate that the rats maintained good performance on both NBN and AM trials following sham and sound exposures.

As expected, following the 15-min sham exposure, the rats correctly identified the quiet trials, whereas the 15-min sound exposure caused all rats to demonstrate tinnitus-positive behavior by shifting their responses for quiet stimuli to the left (NBN) trough (Two-way RM ANOVA, significant interaction for time and exposure, *F*_1,9_ = 64.573, *p* < 0.001) (Figures 2E, F). These results are consistent with studies conducted in human subjects in which a brief exposure to loud noise results in the immediate onset of acute tinnitus (Loeb and Smith, 1967; Atherley et al., 1968). On average, sound-exposed rats mistakenly identified 39.1 ± 3.7% of quiet trials as NBN during behavioral testing, whereas the same rats only misidentified 7.0 ± 2.2% of quiet trials following the sham exposure.

### 3.2. Chronic tinnitus induced by a 60-min intense sound exposure

In order to determine whether the 2AFC behavioral paradigm could be used to detect the presence of chronic tinnitus induced by a 60-min intense sound exposure, we first carried out pilot experiments to identify an appropriate time point post-exposure in which we could run animals on the testing protocol. As shown in Figure 3A, performance on quiet trials remained consistent when rats had one full week off between training and testing on the behavioral paradigm [one-tailed paired t-test on baseline vs one week off, *t*(9) = 1.687, *p* > 0.05]. However, when rats were given two full weeks off between training and testing, their misidentification of quiet trials significantly increased, indicative of a false-positive screening of tinnitus in some of the animals [one-tailed paired t-test on baseline vs. two weeks off, *t*(9) = 2.7, *p* < 0.05]. Similarly, after having one week off from training, rats could only be run on the testing protocol up to two days in a row before a significant increase in the misidentification of quiet trials occurred [One-way ANOVA, *F*_4,32_ = 2.701, *p* < 0.05] (Figure 3B). Based on these results, we opted to assess our rats for the presence of chronic tinnitus one week after the intense sound exposure without running them on the testing protocol multiple days in a row.

A separate cohort of rats (*n* = 10) were trained on the behavioral paradigm to distinguish between quiet, AM noise and NBN stimuli, and were subsequently given 60-min sham and sound exposures. As expected from our pilot testing, rats could still reliably identify AM, NBN, and quiet trials post-sham exposure, even after one week off between the sham exposure and testing day (Figures 4A, C, E). Following the sound exposure, a significant increase in the number of misidentified AM trials was observed; however, the rats were still able to correctly identify > 80% of AM trials (Two-way RM ANOVA, significant interaction for time and exposure, *F*_1,9_ = 12.798, *p* < 0.01) (Figures 4C, D). Furthermore, the sound exposure caused a significant decrease in NBN performance (68.4 ± 8.8% correct post-sound vs. 96.9 ± 1.3% correct post-sham; Two-way RM ANOVA, significant interaction for time and exposure, *F*_1,9_ = 10.434, *p* < 0.01) (Figures 4A, B). Not surprisingly, a wide range in NBN performance was observed across animals post-sound exposure, with 5 out of the 10 rats scoring > 80% correct, and the remaining 5 out of 10 rats scoring between 15 and 68% correct post-sound exposure. In addition to the changes in NBN performance post-sound exposure, there was a significant increase in the number of quiet trials misidentified as NBN, indicative of tinnitus-positive behavior (36.1 ± 5.9% misidentified post-sound vs. 5.1 ± 0.8% misidentified post-sham; Two-way RM ANOVA, significant interaction for time and exposure, *F*_1,9_ = 27.875, *p* < 0.01) (Figures 4E, F). Unlike the results from the first experimental series in which all rats screened positive for acute tinnitus by way of a significant increase in the number of misidentified quiet trials immediately after the 15-min sound exposure, not all rats demonstrated evidence of chronic tinnitus one week after the 60-min sound exposure.

When each rat’s performance on NBN trials was plotted versus their quiet trial performance, no significant relationship was observed, indicating that an animal’s performance on NBN trials was not predictive of their performance on quiet trials (Figure 5A). This point is highlighted by the fact that rats which demonstrated the greatest decline in NBN performance post-sound exposure did not demonstrate the greatest increase in misidentified quiet trials (e.g., Rat A in Figures 4B, F, 5). Similarly, some of the rats that demonstrated the greatest increase in misidentified quiet trials post-sound exposure still scored > 80% correct on NBN trials (e.g., Rats H and G in Figures 4B, F, 5). This demonstrates that the changes in behavioral performance following the intense sound exposure were specific to the quiet condition, and were not related to a change in overall behavioral performance. As expected, rats exposed to intense sound (12 kHz tone, 120 dB SPL, 1 h) had a high-frequency hearing loss, characterized by an average post-sound exposure threshold of 66.5 ± 7.6 dB at 20 kHz, and by thresholds of 31.5 ± 2.6 dB and 40.5 ± 1.4 dB for the 4 kHz and click stimuli, respectively. Similar to the results depicted in Figure 5A, which plots each rat’s performance on NBN trials versus their performance on quiet trials, no clear relationship was observed between the level of high-frequency hearing loss for each rat and their performance on quiet trials post-sound exposure (Figure 5B). For example, Rats I and J both screened negative for tinnitus—as evidenced by no significant change in their quiet trial performance post-sound exposure (Figure 4E; filled red squares)—but these rats had dramatically different high frequency hearing thresholds resulting from the same sound exposure (Figure 5B).

## 4. Discussion

A reliable behavioral paradigm is essential when using animal models to investigate the neural mechanisms underlying tinnitus. In our previous publication (Stolzberg et al., 2013), we reported a novel two-alternative categorization task optimized for identifying acute drug-induced tinnitus with simultaneous recordings of neural activity in behaving rats. Here, we provide further validation of our previously established paradigm in its effectiveness at assessing rats for acute and chronic sound induced tinnitus. As discussed in detail below, validation of our paradigm is supported by: (1) its resistance to false-positive screening of rats for intense sound-induced tinnitus, and (2) its ability to screen individual animals in order to identify the variabilities in tinnitus development and hearing loss following intense sound exposure. In light of these findings, our paradigm would be useful for investigations into the efficacy of novel therapeutics for tinnitus, as well as studies seeking to uncover the putative neural mechanisms of tinnitus. However, several considerations and limitations of the paradigm should be addressed when using our model for studying chronic sound-induced tinnitus, as discussed in detail below.

### 4.1. Is the 2AFC behavioral paradigm resistant to falsely-screening rats for tinnitus?

When validating an animal behavioral paradigm, it is important to consider whether false-positive screenings can occur when assessing the presence of tinnitus. To that end, we carried out a number of important control experiments in order to confirm that the behavioral screening following intense sound exposure was indeed representative of tinnitus and not the result of a separate confounding factor. To validate the use of our paradigm for the assessment of acute tinnitus, rats received 15-min sham and sound exposures immediately prior to behavioral testing. Sham exposures were not expected to cause tinnitus in rats, and this was indeed reflected in our findings, as there were no significant group differences in performance on the quiet, AM, or NBN trials following sham exposure (blue symbols in Figures 2A, C, E). Similar behavioral profiles were observed in our previous study when rats were given systemic injections of saline as a sham condition (Stolzberg et al., 2013). Furthermore, when performance of individual animals was assessed, only a few rats had positive z-scores for quiet trial performance post-sham exposure, demonstrating that the possibility of false positive results for our paradigm are low.

Before assessing chronic tinnitus, we first determined the appropriate time series following intense sound exposure in which animals should be run on the testing protocol, given the potential for task performance to be influenced by repetitive test sessions or a long layoff following sound exposure. As shown in Figures 3A, B, when control rats were given more than one week off between training and testing, or they were run on the testing protocol more than two days in a row, their performance on quiet trials began to falsely indicate the presence of tinnitus. With these results in mind, we determined that the best time to assess the presence of chronic tinnitus was one week post-exposure. These findings highlight the importance of selecting appropriate testing days when evaluating the presence of chronic tinnitus. Similar to the results obtained when screening rats for acute tinnitus with our paradigm, and consistent with our pilot testing, sham exposures had no significant effect on group performance of the quiet, AM, or NBN trials one week later (Figures 4A, C, E). Importantly, these extensive sham experiments allowed us to be confident that the paradigm was able to correctly classify control rats as *not* having tinnitus. Moreover, the consistency of the results following sham exposures emphasizes the robustness of our behavioral paradigm in its resistance to false indications of acute or chronic tinnitus; a criterion that is essential for any successful behavioral model of tinnitus.

### 4.2. Variable outcomes following intense sound exposure: The relationship between hearing loss and chronic tinnitus?

A challenge of studying sound-induced tinnitus, regardless of the behavioral paradigm used, is the potential for considerable variability in outcomes across animals; findings which may arise due to differing degrees of hearing loss induced by a given sound exposure. Furthermore, the requirement of different animal cohorts for control and experimental series has been a considerable drawback of previously established shock avoidance tinnitus models, as it is well-known that tinnitus in humans is highly variable at the level of the individual. Thus, we considered the utility of our paradigm to screen for chronic tinnitus in individual sound-exposed rats that experienced variable levels of permanent hearing impairment.

Following the intense sound exposure, not all rats demonstrated evidence of chronic sound-induced tinnitus. Consistent with our results, it is well-established that not all subjects exposed to the same level of excessive sound will develop tinnitus. For example, previous behavioral work by Brozoski et al. (2007) showed that a one-hour exposure to 120 dB SPL band-limited noise did not induce tinnitus-like behavior equally in all rodents. Variable tinnitus behavioral profiles were also observed in individual rats following noise exposure using the behavioral paradigm developed by Sederholm and Swedberg (2013), as well as the one developed by Heffner and Harrington (2002). Moreover, human studies have revealed that of the number of returning war veterans surveyed who were exposed to blast trauma (a severe form of noise exposure), only 49% of them went on to develop tinnitus (Cave et al., 2007). Thus, in the present study, it was not surprising that not all rats showed behavioral evidence of chronic tinnitus in the week following intense sound exposure.

In addition to the variable outcome of chronic tinnitus induction, we also observed variability in NBN performance one week following sound exposure (Figures 4A, B). The post-exposure decline in NBN performance led us to postulate that some rats likely developed a hearing loss that prevented them from perceiving NBNs, and as such, they mistakenly probed the feeder trough associated with the quiet stimulus during NBN trials. Not surprisingly, variable levels of high-frequency hearing loss were also observed following sound exposure. These results are consistent with previous findings in other models of noise-induced hearing loss in which considerable inter-animal variability was observed following exposure to the same acoustic trauma (Cody and Robertson, 1983; Mulders et al., 2011).

It is often suggested that a strong link exists between hearing loss and the presence of tinnitus, as the majority of patients who suffer from tinnitus have some degree of measurable hearing impairment (Axelsson and Ringdahl, 1989; Henry et al., 2014). That said, some tinnitus sufferers are suspected of having “hidden hearing loss”; i.e., while they have normal audiometric hearing thresholds, they still have cochlear damage characterized by a reduction in sound-evoked activity of their auditory nerve fibers (Schaette and McAlpine, 2011). In the present study, although we did not design our sound exposure protocol to cause hidden hearing loss, we did observe varying degrees of high-frequency hearing impairment in the rats that screened positive for tinnitus. Moreover, similar to Heffner and Harrington (2002) who used a shock avoidance behavioral paradigm to identify tinnitus following varying durations of sound exposure, we also observed that rats with similar degrees of hearing loss did not all screen positive for tinnitus. As discussed above, this finding is not surprising, as many individuals with hearing loss do not experience tinnitus. Finally, we also observed varying hearing loss in rats that screened negative for tinnitus following sound exposure; e.g., one of the “no tinnitus” rats had limited high frequency hearing impairment (ABR threshold: 15 dB SPL at 20 kHz), whereas another rat had a severe hearing loss (ABR threshold: 80 dB SPL at 20 kHz). Looking forward, we envision using our 2AFC task coupled with simultaneous neural recordings to screen for tinnitus in rats with hidden hearing loss, as this would ultimately provide an effective platform to test theories derived from recent computational modeling studies and review articles that consider the relationship between (hidden) hearing loss and tinnitus (Schaette and McAlpine, 2011; Schaette and Kempter, 2012; Zeng, 2020).

### 4.3. Experimental considerations

The results of the present study highlight the importance of a number of experimental considerations to take into account when using our 2AFC paradigm to screen rodents for sound-induced tinnitus. First, extensive pilot testing and sham exposure experiments demonstrate that our paradigm is limited by the number of times that individual animals can be screened for the presence of chronic tinnitus post-sound exposure (Figures 3A, B). Based on these findings, we recommend that rodents are only run on the testing protocol up to two days in a row post-sound exposure, and that animals are given no more than one week off between sound exposure and the testing protocol. It is important to note that repeatedly running rats on the testing protocol, in which they are no longer punished or rewarded for their quiet trial performance, may lead to random selection of either food trough over time during quiet trials. However, our sham and pilot testing clearly demonstrate that the misidentification of quiet trials as NBN when the animals are first run on the testing protocol post-sound exposure reflects the presence of tinnitus and not a false-positive screening due to random probing of the food troughs.

A major benefit of our 2AFC paradigm is the ability to screen individual animals for the presence of tinnitus, as opposed to solely analyzing group behavioral performance; an important feature given that not all sound-exposed animals may develop tinnitus. However, it is important to determine an appropriate method of identifying which animals have tinnitus based on their behavioral performance. In the present study, we adopted a z-score analysis similar to that used in previous tinnitus publications (Heffner, 2011; Stolzberg et al., 2013), and classified tinnitus-like behavior as a significant decrease in responses to the correct feeder trough during quiet trials on the post- sham or sound testing day compared to the response rate on quiet trials during the preceding 5 baseline days. When using this approach, the “floor effect” must be considered, by which holding animals to a very high baseline performance criteria (i.e., 92% in the present study) can result in a very minor change in behavioral performance post-sham or sound exposure leading to a significant z-score from baseline. For example, in Figure 4E, two rats showed significant z-scores during quiet trial performance post-sham exposure (open blue circles) despite a very minor change in their behavioral performance from pre-sham baseline testing. Potential ways to address this concern in the future would be to hold animals to a less strict performance criterion (i.e., ∼85%) at baseline, or to set a standard threshold in performance across rats so that any individual rat whose performance crosses the threshold will be classified as having tinnitus.

Additional considerations should be made when identifying cases in which it may be inappropriate to include an animal for analysis of sound-induced tinnitus based on their behavioral performance. For example, if an animal shows a dramatic change in performance post-sham exposure, we would first suggest that the sham exposure and testing be repeated. If the animal still demonstrates a significant change in performance following the second sham exposure, we would recommend that the animal be excluded from further analysis (i.e., the animal should not go on to be sound exposed, and then tested for sound-induced tinnitus). Furthermore, if an animal shows an overall change in performance on all trial types (NBN, AM, and quiet) post-sound exposure, suggesting that the rat is no longer performing the task appropriately, we would recommend that the animal be excluded from analysis. Related to this, particular attention should be given to identifying animals that display a right or left side bias during testing (i.e., animals that go to the left feeder trough for the majority of trials, regardless of trial type). For example, Rat F in Figure 4 falls into this category by demonstrating significant impairments across all trial types post-exposure during testing for chronic tinnitus (the rat displays a potential left side bias by choosing the left feeder trough for all trial types, including AM trials). Similarly, Rat A in Figure 4 may display a right side bias post-exposure, as evidenced by selection of the right feeder trough for all trial types. While the need for animal exclusion is quite rare when using the present paradigm, it is an important factor to consider in order to avoid falsely-screening animals for the presence of tinnitus. Potential ways to address this concern in the future would be to set a standard threshold in performance across rats for NBN and AM trials so that any individual rat that demonstrates significantly impaired performance on these trial types should be considered as having a potential side bias, and their performance on quiet trials should be interpreted with caution. Finally, in an effort to limit potential side biases that may emerge due to the rats’ difficulty hearing the task stimuli, another experimental consideration could be to adjust the stimulus intensities for each rat according to its ABR-confirmed sensation level rather than a set 75 dB SPL, as this customization could better accommodate inter-animal variability in hearing loss.

Given that we first used our 2AFC task to screen rats for salicylate-induced tinnitus (Stolzberg et al., 2013), it is worth comparing those findings with the present study in which acute tinnitus was induced by brief yet intense sound exposure. Consistent with the behavioral profile observed following salicylate administration, the rats exposed to a 12 kHz tone at 110 dB SPL for 15 min showed no change in performance during the NBN trials, but did show a significant increase in the number of misidentified quiet trials; findings indicative of the presence of tinnitus in both models. In the present study, we also observed a slight, but significant increase in the misidentification of AM trials for some rats following sound exposure. This is likely due to the presence of hearing loss which could interfere with the processing of temporal cues (Tyler et al., 1982; Henderson et al., 1984; Radziwon et al., 2019). Interestingly, we did not see this change in AM performance following salicylate exposure in our previous study (Stolzberg et al., 2013), perhaps because of the disparate effects that salicylate and noise exposure have on the auditory periphery (Henderson et al., 2006; Stolzberg et al., 2012).

### 4.4. Future directions

Now that we have confirmed the validity of our paradigm and its resistance to false-positives, we can envision future studies using this screening tool to investigate novel therapeutics for tinnitus prevention, as well as the pathophysiology of tinnitus. For example, because our behavioral paradigm was sensitive enough to reveal animals with differing post-exposure profiles (i.e., not all rats had tinnitus), future studies could expose groups of rats to loud sound, followed by administration of either a therapeutic-of-interest or a vehicle-control, and ultimately determine the proportion of rats in each group that go on to screen positive for tinnitus. As there is currently no widely accepted drug treatment for tinnitus prevention, and many clinical trials seeking to alleviate chronic tinnitus have found that only a subset of subjects within the treatment group experience benefit (Allman et al., 2016), it will be worthwhile for future animal models to consider the ratio of “responders” versus “non-responders” following a given intervention.

One of the major advantages of using animal models to investigate the neural basis of tinnitus is the potential to perform longitudinal studies in which a given animal’s brain activity can be compared before versus after induction of tinnitus via intense sound exposure. In addition to such within-subject comparisons, efforts to contrast the electrophysiological recordings in tinnitus-positive versus tinnitus-negative rats that showed similar hearing loss profiles post-exposure would be expected to provide valuable insight into the neural correlates of tinnitus, as this comparison would be freed from issues related to hearing loss alone. This comparative approach could also be strengthened by including a complementary electrophysiological investigation between groups of animals that both screened positive for tinnitus yet differed in their degree of hearing loss, as this would help to unravel the seemingly complex relationship between the effect of hearing loss and/or tinnitus on brain plasticity. Related to the varying degrees of hearing loss observed in tinnitus patients, computational studies have attempted to model various neural mechanisms thought to underlie tinnitus, such as changes in lateral inhibition (Gerken, 1996), gain adaptation (Parra and Pearlmutter, 2007), homeostatic plasticity (Schaette and Kempter, 2006, 2009; Chrostowski et al., 2011), as well as increased central noise and variance (Zeng, 2013, 2020). Given that many of these computational models describe an increase in spontaneous firing rates as a neural correlate of tinnitus (reviewed by Schaette and Kempter, 2012), it would be worthwhile for future studies to use our 2AFC task (with its emphasis on a having rats attend to their tinnitus during actual quiet trials) to assess whether the rate and/or synchronization of the spontaneous spiking activity in a given trial does indeed predict whether a rat will go on to report that it perceives a steady sound (i.e., tinnitus).

Overall, the aforementioned examples of within-subject as well as between-subject comparisons are well-suited to the 2AFC behavioral paradigm, as we have previously confirmed that it is possible to simultaneously record neural activity at the very moments when the rats are being screened for behavioral evidence of tinnitus (Stolzberg et al., 2013). Related to this important feature, we foresee future studies being able to use this behavioral paradigm in combination with advanced techniques for real-time manipulation (e.g., optogenetics; chemogenetics) or monitoring (e.g., genetically-encoded calcium indicators) of cell/circuit-specific activity underlying sensory perception. Ultimately, given the ever-increasing number of transgenic rat models available, it is reasonable to propose that these techniques could assist in uncovering the neural signature of tinnitus, and we suggest that our behavioral paradigm could offer a suitable platform for such investigations.

## Data availability statement

The raw data supporting the conclusions of this article will be made available by the authors, without undue reservation.

## Ethics statement

This animal study was reviewed and approved by the University of Western Ontario Animal Care and Use Committee and all procedures were in accordance with guidelines established by the Canadian Council of Animal Care.

## Author contributions

SH, KB, MT, and AS: data collection and analysis. All authors: project conceptualization, data interpretation, and manuscript writing and editing.
