# Supplementary material for: Using an appetitive operant conditioning paradigm to screen rats for tinnitus induced by intense sound exposure: Experimental considerations and interpretation
Source: Front Neurosci. 2023 Feb 10;17:1001619. doi: 10.3389/fnins.2023.1001619 (PMC9950262; doi:10.3389/fnins.2023.1001619)
Supplement: Supplementary file 1 [file Table_1.docx]

**Table 1:** Behavioural training steps for the 2AFC task.

| **Protocol** | **Left Trough Stimuli** | **Right Trough Stimuli** | **Reward Rate** | **Description** |
| --- | --- | --- | --- | --- |
| Phase 1 | NBN 16 | Quiet | 100% (plus automatic reward) | Introduce nose poke, feeder troughs and auditory stimuli |
| Phase 2A | NBN 16 | Quiet | 100% | Associate left and right feeder troughs with appropriate stimuli |
| Phase 2B | NBN 8, 12, 16, 20, 24 | Quiet | 100% | Generalization of NBN stimuli |
| Phase 2C | NBN 8, 12, 16, 20, 24 | Quiet, AM | 100% | Introduce AM stimuli |
| Final Training | NBN 8, 12, 16, 20, 24 | Quiet, AM | 70% | Introduce reduced reward rate to prepare for test days when Quiet trials are not rewarded |
| Testing Day | NBN 8, 12, 16, 20, 24 | Quiet, AM | 90% for NBN and AM; 0% for Quiet | Quiet trials no longer punished or rewarded; Identification of tinnitus positive behavior |
